# Globalized simulation-driven miniaturization of microwave circuits by means of dimensionality-reduced constrained surrogates

**DOI:** 10.1038/s41598-022-20728-0

**Published:** 2022-09-30

**Authors:** Slawomir Koziel, Anna Pietrenko-Dabrowska, Marzieh Mahrokh

**Affiliations:** 1 Engineering Optimization & Modeling Center, Reykjavik University, 102 Reykjavik, Iceland; 2 Faculty of Electronics, Telecommunications and Informatics, Gdansk University of Technology, 80-233 Gdansk, Poland

**Keywords:** Electrical and electronic engineering, Computational science

## Abstract

Small size has become a crucial prerequisite in the design of modern microwave components. Miniaturized devices are essential for a number of application areas, including wireless communications, 5G/6G technology, wearable devices, or the internet of things. Notwithstanding, size reduction generally degrades the electrical performance of microwave systems. Therefore, trade-off solutions have to be sought that represent acceptable compromises between the ability to meet the design targets and physical compactness. From an optimization perspective, this poses a constrained task, which is computationally expensive because a reliable evaluation of microwave components has to rely on full-wave electromagnetic analysis. Furthermore, due to its constrained nature, size reduction is a multimodal problem, i.e., the results are highly dependent on the initial design. Thus, utilization of global search algorithms is advisable in principle, yet, often undoable in practice because of the associated computational expenses, especially when using nature-inspired procedures. This paper introduces a novel technique for globalized miniaturization of microwave components. Our technique starts by identifying the feasible region boundary, and by constructing a dimensionality-reduced surrogate model therein. Global optimization of the metamodel is followed by EM-driven local tuning. Application of the domain-confined surrogate ensures low cost of the entire procedure, further reduced by the incorporation of variable-fidelity EM simulations. Our framework is validated using two microstrip couplers, and compared to nature-inspired optimization, as well as gradient-based size reduction. The results indicate superior miniaturization rates and low running cost, which make the presented algorithm a potential candidate for efficient simulation-based design of compact structures.

## Introduction

Design of contemporary microwave passive circuits is a non-trivial endeavour. Performance and functionality demands have been continuously growing to satisfy the needs of the emerging application areas such as mobile communications^1^, internet of things^2^, remote sensing^3^, microwave imaging^4^, energy harvesting^5^, autonomous vehicles^6^, or implantable device^7^. Some of the requirements include multi-band operation^8^, reconfigurability^9^, harmonic suppression^10^, or custom phase characteristics^11^. Furthermore, many applications impose constraints on the physical size of the devices, which fosters miniaturization^12–15^. Miniaturization is essentially a two-stage process. Initially, a basic circuit architecture is selected to ensure compact dimensions^16,17^, often with the use of techniques such as transmission line (TL) folding/meandering^18^, utilization of the slow-wave phenomenon^19^ (typically, in the form of compact microwave resonant cells, CMRCs^20^), multi-layer realizations^21^, or incorporation of various supplementary components (stubs^22^, defected ground structures^10^, substrate-integrated waveguides^23^, shorting pins^24^). All of these methods result in geometrically complex structures, whose accurate evaluation requires full-wave electromagnetic (EM) analysis due to the presence of cross-coupling effects in densely arranged circuit layouts. At the same time, geometrical modifications lead to the increase of the number of parameters that have to be simultaneously tuned in order to control both the circuit size and electrical figures of merit. As size reduction is detrimental to electrical performance of the system, any practical design is a trade-off between compactness and functionality. Initial circuit dimensions can usually be obtained using a combination of equivalent networks and parametric studies, yet rigorous numerical optimization is indispensable to significantly enhance the system performance.

Nowadays, parameter tuning is more and more often carried out using rigorous numerical optimization methods, which is recommended due to their ability to handle multiple parameters, objectives and constraints^25–27^. Optimization is not only used for the purpose of design closure (final tuning of geometry parameters, often using local algorithms^28^), but also multi-criterial design^29^, uncertainty quantification (tolerance analysis^30^, design centering^31^), and global optimization^32^. Whatever the purpose, microwave circuit optimization is a challenging endeavor. Perhaps the most significant bottleneck is its high computational cost when executed at the level of EM simulation models, otherwise necessary to ensure reliability of the process. While the costs are often manageable in the case of local (e.g., gradient-based) tuning, global or multi-objective optimization, as well as statistical design, are considerably more demanding^33,34^. Consequently, there have been numerous techniques developed to improve computational efficiency of EM-driven optimization. Some of these methods include utilization of adjoint sensitivities^35,36^, restricted sensitivity updates^37–39^, the employment of (fast) dedicated solvers^40^, mesh deformation approaches^41^, feature-based optimization^42^, or cognition-driven design^43^. Yet, one of the most important developments in making simulation-based design more practical in terms of CPU expenses, has been the incorporation of surrogate modeling methodologies^44–47^.

Surrogate-assisted optimization (SBO) has attracted a considerable attention in the design of high-frequency circuits, including microwave and antenna components, primarily because of its ability to accelerate simulation-based procedures, including local^48^, and global optimization^49^, robust design^50^, or multi-criterial optimization^51^. Surrogate-assisted procedures utilize both data-driven^52^ or physics-based metamodels^53^. Data-driven techniques are versatile and readily transferrable between the problem domains^54^, which make them the most popular class of modeling methods. Specific approaches often employed in the context of high-frequency engineering include kriging^55^, radial basis functions^56^, many variations of artificial neural networks^57–59^, support vector regression^60^, Gaussian process regression^61^, or polynomial chaos expansion (PCE)^62^. Data-driven models are cheap to evaluate, but they are affected by the curse of dimensionality: the number of training data samples necessary to construct reliable models quickly grows with the number of parameters and parameter ranges, and may become unmanageable even for medium-size problems. Physics-based surrogates are constructed using a lower-fidelity representation of the system of interest (e.g., equivalent network^63^, or coarse-discretization EM analysis^64^). The problem-specific knowledge embedded in the low-fidelity model enhances generalization capability of the surrogates of this class^65^. At the same time, it limits the applicability range because each problem requires its own low-fidelity model. Some of popular techniques include space mapping^66^, and response correction methods^67–69^, most of which are typically used for local optimization purposes. Surrogate-assisted frameworks allowing for solving expensive constrained optimization problems have been recently proposed in^70^ and^71^.

As mentioned earlier, size reduction constitutes a prerequisite in the design of contemporary microwave components. It is normally addressed at the level of selecting the circuit architecture^72–74^, yet appropriate parameter tuning plays just as important part. From numerical perspective, size reduction is a constrained task with expensive constraints that require evaluating through EM analysis (e.g., acceptance thresholds imposed on the circuit bandwidth, power split ratio, or phase responses)^75^. As size reduction is detrimental to electrical performance, at least some of the constraints remain active at the optimal solution, which emphasizes the role of feasible region boundary exploration in the search process^76^. These challenges can be addressed by implicit constraint handling using a penalty function approach^77^, where the problem is reformulated into a formally unconstrained one. However, performance of the optimization process turns out to be contingent upon the appropriate choice of penalty coefficients^78^, which are normally selected by trial and error. This gave rise to adaptive penalty coefficient strategies^79,80^. Recently, explicit constraint handling methods have been proposed^81^, along with the techniques for customized treatment of equality constraints, based on correction procedures^82^. Another approach to constraint handling in the context of design of antennas and antenna arrays using evolutionary algorithms that allows for circumventing the issue of an appropriate setting of the penalty coefficients, has been proposed in^83,84^.

The optimization techniques outlined in the previous paragraph are local search procedures, which are highly dependent on the available starting points. At the same time, miniaturized microwave components are often developed using transmission line meandering^85^, CMRCs^20^, or various geometrical modifications^86,87^, which leads to parameter redundancy (e.g., a typical number of geometry parameters of CMRC is four to six versus two for a conventional TL). The increased number of degrees of freedom enables the necessary flexibility; yet, its handling calls for global optimization. Conventional global search methods (e.g., nature-inspired population-based algorithms^88,89^) are not suitable for the purpose due to poor computational efficiency. This work proposes a novel procedure for globalized miniaturization of passive microwave circuits, which is designed to lessen the cost of the search process while maintaining reliability. The presented technique is a multi-stage process, which starts by (roughly) approximating the feasible region boundary using a set of randomly generated parameter vectors coupled with initial (local) optimization runs. Subsequently, a reduced-dimensionality domain is established in the feasible boundary region, along with a fast surrogate model, the latter utilized to conduct a globalized size reduction. The search process is concluded by final miniaturization-oriented parameter tuning of the circuit. The abovementioned dimensionality reduction is achieved using the spectral analysis of the pre-optimized parameter vectors. The initial steps of the search process are executed using low-fidelity model to lower the CPU cost even further. Our methodology has been validated using a compact rat-race coupler and a dual-band power divider. The numerical results demonstrate superior performance of the proposed routine, with regard to both the computational efficiency and reliability, as well as constraint control, as compared to the nature-inspired optimization and multiple-start local search.

The primary technical contributions of the paper can be summarized as follows: (i) the development of a novel framework for globalized EM-driven miniaturization of passive microwave circuits, which incorporates several mechanisms (variable-fidelity EM analysis, surrogate modelling, and dimensionality reduction), (ii) a demonstration of the competitive performance of the presented method as compared to the state-of-the-art methods (both local and global), also in terms of achievable miniaturization rates, (iii) a demonstration of the search process reliability, especially low variance of the optimization results (equivalent to consistent repeatability). According to the authors’ knowledge, the literature does not offer any size-reduction framework featuring comparable properties and performance. Consequently, the proposed approach may become an interesting alternative to existing methods, particularly in terms of combining computational efficiency and achievable miniaturization rates.

## Globalized EM-driven miniaturization using variable-fidelity models and spectral analysis

This section provides the details of the globalized optimization procedure for passive microwave components introduced in the paper. The EM-driven size reduction problem is formulated in "EM-driven size reduction: problem statement" Section. The concept of the optimization algorithm is described in Globalized size reduction: explanation of the concept Section. "Feasible Region boundary approximation" Section elaborates on feasible region boundary approximation, one of the keystones of the presented methodology. The surrogate modeling stage is outlined in "Surrogate model construction",  "Surrogate model optimization for size reduction" Sections. delineates global optimization of surrogate model, whereas "Final parameter adjustment" Section discusses the final (gradient-based) design closure. The entire optimization framework is summarized in "Globalized EM-driven size reduction: complete procedure" Section using a pseudocode and a flow diagram.

### EM-driven size reduction: problem statement

Design of compact microwave components consists of the two major stages: (i) a selection of the circuit geometry, and (ii) parameter tuning. The first stage is essential to ensure structural miniaturization (e.g., by replacing TLs with their abbreviated counterparts such as CMRCs^90^), whereas the second allows for exploring further the size reduction potential of the chosen architecture, in particular, to push the design as much as possible towards feasible region boundary, where the electrical performance parameters are barely satisfied in exchange for additional reduction of the circuit physical dimensions.

In the following, we will use ***x*** = [*x*_1_ … *x*_*n*_]^*T*^ for a vector of design variables, and by *A*(***x***) the circuit size (e.g., footprint area). The miniaturization problem is simply stated as

1 $$ {\mathbf{x}}^{*} = \arg \mathop {\min }\limits_{{{\mathbf{x}} \in X_{f} }} A({\mathbf{x}}) $$

where *X*_*f*_ is a feasible space, i.e., the region in which all design constraints are satisfied. The constraints can be of inequality type, *g*_*k*_(***x***) ≤ 0, *k* = 1, …, *n*_*g*_ (e.g., acceptance threshold for the circuit operating bandwidth), and equality constraints *h*_*k*_(***x***) = 0, *k* = 1, …, *n*_*h*_ (e.g., target power split ratio).

The constraints imposed on electrical characteristics of the circuit are expensive to evaluate (require EM simulation). Consequently, their explicit handling might be problematic, although some recent strategies demonstrated promising results (e.g.^81^,). A convenient alternative is implicit handling using a penalty functions^77^. According to this approach, the original objective function is supplemented by scaled constraint violations. We have

2 $$ {\mathbf{x}}^{*} = \arg \mathop {\min }\limits_{{\mathbf{x}}} U({\mathbf{x}}) $$

where the merit function *U* is given by

3 $$ U({\mathbf{x}}) = A({\mathbf{x}}) + \sum\nolimits_{k = 1}^{{n_{g} + n_{h} }} {\beta_{k} c_{k} ({\mathbf{x}})} $$

The second term in (3) consist of penalty functions *c*_*k*_(***x***) and proportionality coefficients *β*_*k*_; *n*_*c*_ = *n*_*g*_ + *n*_*h*_ is the overall number of constraints. Table 1 provides a few examples of constraints that may be encountered in size reduction tasks. Table 2 shows example definitions of the penalty functions, often expressed through relative violations. It should be noted that the formulation (2), (3) corresponds to soft constraint handling, i.e., it does not guarantee their exact satisfaction. As a matter of fact, the constraint control is reliant on coefficients *β*_*k*_, which should be adjusted appropriately. Too low values result in an insufficient control over constraint violations, whereas the values that are too high lead to numerical problems as the objective function becomes very steep at the feasible region boundary. This issue has been addressed by adaptive coefficient adjustment schemes^78,80^, where the values of *β*_*k*_ are changed based on currently-detected violations, as well as the algorithm convergence status^78^.

**Table 1:** Example constraints in size-reduction of microwave components.

Constraint	Type	Analytical description^$^
Input matching |*S*_11_| not exceeding –20 dB over the operating bandwidth[*f*_1_ *f*_2_]	Inequality	|*S*_11_(***x***,*f*)|≤ − 20 dB for *f* $$\in$$ [*f*_1_ *f*_2_]
Port isolation |*S*_41_| not exceeding –20 dB over the operating bandwidth [*f*_1_ *f*_2_]	Inequality	|*S*_41_(***x***,*f*)|≤ − 20 dB for *f* $$\in$$ [*f*_1_ *f*_2_]
In-band transmission ripple not exceeding 0.2 dB over the operating bandwidth [*f*_1_ *f*_2_]	Inequality	|*S*_21_(***x***,*f*)|≥ − 0.2 dB for *f* $$\in$$ [*f*_1_ *f*_2_]
Power split ratio between output ports 2 and 3 equal to *K*_*P*_ at the center frequency *f*_0_	Equality	|*S*_31_(***x***,*f*)|–|*S*_21_(***x***,*f*)|= *K*_*P*_ at *f* = *f*_0_;
Phase difference between output ports 2 and 3 equal to 90° at the center frequency *f*_0_	Equality	∠*S*_31_(***x***,*f*)–∠*S*_21_(***x***,*f*) = 90° at *f* = *f*_0_;

^$^The symbol |*S*_*jk*_(***x***,*f*)| stands for the modulus of the *S*-parameter *S*_*jk*_ at the design ***x***, and frequency *f*.

**Table 2:** Possible formulation of penalty functions for constraints of Table 1.

Constraint	Penalty function
Input matching |*S*_11_| not exceeding − 20 dB over the operating bandwidth [*f*_1_ *f*_2_]	$c({\mathbf{x}}) = \left[ {\frac{{\max \left\{ {\max \{ f_{1} \le f \le f_{2} :|S_{11} ({\mathbf{x}},f)|\} + 20,0} \right\}}}{20}} \right]^{2}$
Port isolation |*S*_41_| not exceeding − 20 dB over the operating bandwidth [*f*_1_ *f*_2_]	$c({\mathbf{x}}) = \left[ {\frac{{\max \left\{ {\max \{ f_{1} \le f \le f_{2} :|S_{41} ({\mathbf{x}},f)|\} + 20,0} \right\}}}{20}} \right]^{2}$
In-band transmission ripple not exceeding 0.2 dB over the operating bandwidth [*f*_1_ *f*_2_]	$c({\mathbf{x}}) = \left[ {\frac{{\max \left\{ { - \min \{ f_{1} \le f \le f_{2} :|S_{21} ({\mathbf{x}},f)|\} - 0.2,0} \right\}}}{0.2}} \right]^{2}$
Power split ratio between output ports 2 and 3 equal to *K*_*P*_ at the center frequency *f*_0_	$c({\mathbf{x}}) = \left[ {|S_{31} ({\mathbf{x}},f_{0} )| - |S_{21} ({\mathbf{x}},f_{0} )| - K_{P} } \right]^{2}$
Phase difference between output ports 2 and 3 equal to 90° at the center frequency *f*_0_	$c({\mathbf{x}}) = \left[ {\angle S_{31} ({\mathbf{x}},f_{0} ) - \angle S_{21} ({\mathbf{x}},f_{0} ) - 90^\circ } \right]^{2}$

### Globalized size reduction: explanation of the concept

Miniaturization of microwave components is typically obtained by appropriate selection of the circuit architecture (line folding^18^, slow-wave phenomenon^19^, defected ground^10^). Any deviation from conventional structures results in increasing the number of geometry parameters, and creating complex relations between those parameter and electrical characteristics, which are often counter-intuitive. From the perspective of optimization as considered in  "EM-driven size reduction: problem statement" section, this leads to multimodal tasks potentially exhibiting a number of local optima. Appropriate treatment of such problems requires global search methods. However, as mentioned in "Introduction" section, conventional algorithms (e.g., population-based metaheuristics^91^) are just too expensive. On the other hand, surrogate-assisted methods^92^ are hindered by dimensionality issues and high-nonlinearity of microwave component responses. This paper proposes an alternative methodology, designed to improve the efficacy of the optimization-based size reduction process, which includes making the search less dependent on the initial design quality as compared to local methods.

The central concept of the proposed technique is the boundary *X*_*b*_ of the feasible region *X*_*f*_. We have the following definitions (here, *X* is the space of design parameters, usually, delimited by lower and upper bounds):

4 $$ X_{f} = \left\{ {{\mathbf{x}} \in X:g_{k} ({\mathbf{x}}) \le 0\;\;{\text{for}}\;\;k = 1, \ldots ,n_{g} \;\;{\text{AND}}\;\;\;h_{k} ({\mathbf{x}}) = 0\;\;{\text{for}}\;\;\;k = 1, \ldots ,n_{h} } \right\} $$

and

5 $$ X_{b}  = \left\{ {\begin{array}{*{20}l}    {{\mathbf{x}} \in X:g_{k} ({\mathbf{x}}) = 0\;\;{\text{for at least one }}k = 1, \ldots ,n_{g} \;\;} \hfill  \\    {{\text{OR}}\;\;\;h_{k} ({\mathbf{x}}) = 0\;\;{\text{for at least one}}\;k = 1, \ldots ,n_{h} } \hfill  \\   \end{array} } \right\} $$

**Figure 1:**
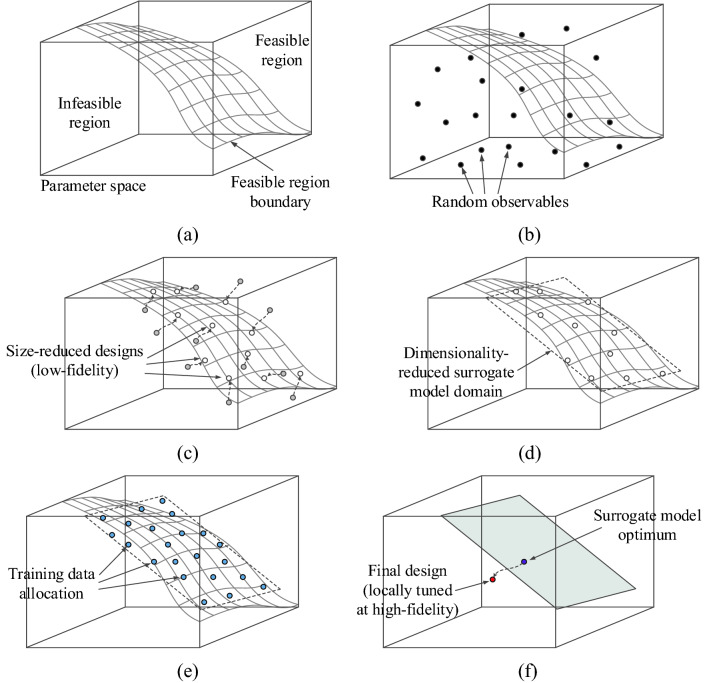
Conceptual illustration of the proposed globalized size reduction procedure involving variable-resolution EM models and dimensionality reduction: (**a**) Exemplary parameter space with feasible and infeasible region indicated along with the boundary region marked in grey, (**b**) Stage 1: allocation of random observables; the acquired EM data will be used to approximate the feasible region boundary, (**c**) Stages 2 and 3: selected observables are optimized for size reduction at low-fidelity EM level, (**d**) Stage 4: spectral analysis of the pre-optimized observables is used to define the domain of the surrogate model in the boundary area, (**e**) Stage 5: training data is allocated in the domain, and kriging interpolation model is constructed, (**f**) Stages 6 and 7: the design obtained through global optimization of surrogate model is finally tuned at high-fidelity level using gradient-based routine.

**Figure 2:**
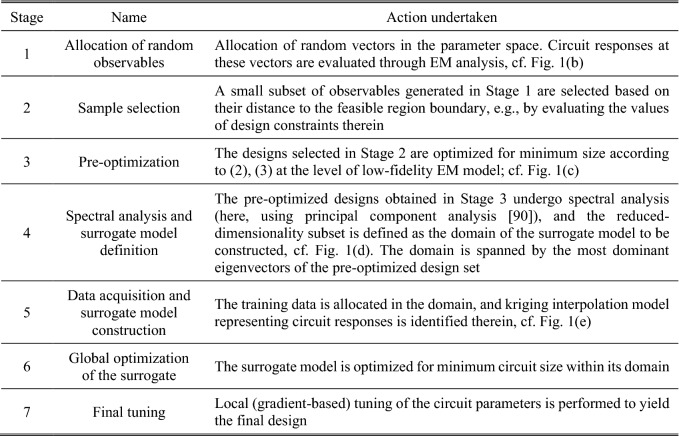
Conceptual stages of globalized size reduction of microwave components.

As miniaturization generally degrades the circuit performance (e.g., reduces the operating bandwidth), minimum-size designs normally reside in *X*_*b*_ as at least one of the constraints is active. Therefore, (approximate) identification of the spatial allocation of *X*_*b*_ allows for narrowing down the part of the parameter space that needs to be explored. The exploration involves surrogate modeling techniques, as well as final EM-driven parameter tuning. Figure 1 shows the overall concepts of the proposed optimization methodology. Figure 2 briefly explains the search stages. Detailed description will be provided in the remaining parts of this section.

To improve computational efficiency of the process, Stages 1 through 3 are carried out using the low-fidelity model ***R***_*c*_, which is based on coarse-discretization EM analysis. At these stages, the accuracy is not of a major concern. Stages 5 and 7 are executed using the high-fidelity model ***R***_*f*_, which is to ensure reliability of the search process. "Feasible region boundary approximation" Section through "Final parameter adjustment" provide the details of how all the stages are implemented. "Globalized EM-driven size reduction: complete procedure" section summarizes the complete framework.

### Feasible region boundary approximation

The parameter space for the microwave circuit of interest is conventionally assumed to be an interval *X* = [***l u***]. Therein, the vectors ***l*** and ***u*** represent the lower and upper parameter bounds. At the component level it may be written as *l*_*k*_ ≤ *x*_*k*_ ≤ *u*_*k*_, *k* = 1, …, *n*. Stages 1 through 3 of the search process (cf. Fig. 2) are arranged as follows. We start by generating *N*_1_ random observables ***x***_*r*_^(*j*)^ that satisfy the following conditions:

- ***x***_*r*_^(*j*)^ ∈ *X* = [***l u***];
- *A*(***x***_*r*_^(*j*)^) ≤ *A*_1_;
- *A*(***x***_*r*_^(*j*)^) ≥ *A*_2_;
- ***x***_*r*_^(*j*)^ satisfy other possible constraints (problem dependent).

Therein, *A*_1_ and *A*_2_ are optional maximum and minimum circuit size values. These might be available from the previous design work with the same circuit, and give the idea of what level of physical sized are achievable for the circuit.

In other words, having such data, we may initially filter out samples that correspond to circuit sizes that are clearly too small or too large. One may also impose additional constraints for the sake of restricting the parameter space regions to be sampled even further. Such constraints should be based on the designer’s knowledge and/or available data. The number of samples *N*_1_ is a user-defined control parameter, typically set to 500.

Having the set of samples, the low-fidelity model is evaluated to obtain the circuit characteristics ***R***_*c*_(***x***_*r*_^(*j*)^), *j* = 1, …, *N*_1_. The best subset of *N*_2_ samples, {***x***_*i*_^(*j*)^}_*j*=1,…,*N*2_ ⊂ {***x***_*r*_^(*j*)^}_*j*=1,…,*N*1_ is selected based on the corresponding values of penalty-based objective function (3). Here, we set *N*_2_ = 20. This number is a reasonable trade-off between the computational cost of subsequent stages and the data on the feasible region boundary *X*_*b*_ that can be obtained therefrom.

The parameter vectors ***x***_*i*_^(*j*)^ are used as initial designs for EM-driven size reduction at the low-fidelity model level. Thus, for *j* = 1, …, *N*_2_, we solve

6 $$ x_{c}^{\left( j \right)} = {\text{ argmin}}\left\{ {x:U\left( x \right)} \right\} $$

Again, *U* is the objective function (3) incorporating the penalty terms. Because the accuracy is not of the fundamental importance at this stage, the problem (6) uses relaxed termination criteria to reduce the CPU cost. In this work, the underlying optimization method is a trust-region (TR) gradient-based algorithm^94^; the circuit response sensitivity is estimated using finite differentiation^95^ (cf. "Final parameter adjustment" section).

Upon solving (6) for *j* = 1, …, *N*_2_, an *N*_3_- element subset of {***x***_*c*_^(*j*)^}_*j*=1,…,*N*2_ is selected that consists of designs being of sufficient quality in terms of constraint violation. This is to filter out designs for which (6) was unsuccessful. Later on, the selected subset will be referred to as {***x***_*c*_^(*j*)^}_*j*=1,…,*N*3_.

### Surrogate model construction

In the proposed global optimization framework, the surrogate model is constructed to represent the circuit responses. As the objective function (3) is a function of these responses, the surrogate-predicted response is employed for its evaluation. Next, global optimization of the surrogate model is carried out, and the approximate design is rendered, which further undergoes a local refinement as shown in Fig. 1f.

The vectors ***x***_*c*_^(*j*)^, *j* = 1, …, *N*_3_, have been obtained by optimizing the circuit for minimum size. Also, due to the definition of the objective function, they exhibit low constraint violations. Consequently, these designs reside in the vicinity of the boundary *X*_*b*_ of the feasible region. Based on {***x***_*c*_^(*j*)^}, we will set up the domain of the surrogate model to be employed for global search purposes. Further, using the spectral analysis of the set {***x***_*c*_^(*j*)^}, the dimensionality of the domain will be reduced as compared to the dimensionality of the original parameter space *X*, which is to limit the computational cost of the surrogate model rendition.

**Figure 3:**
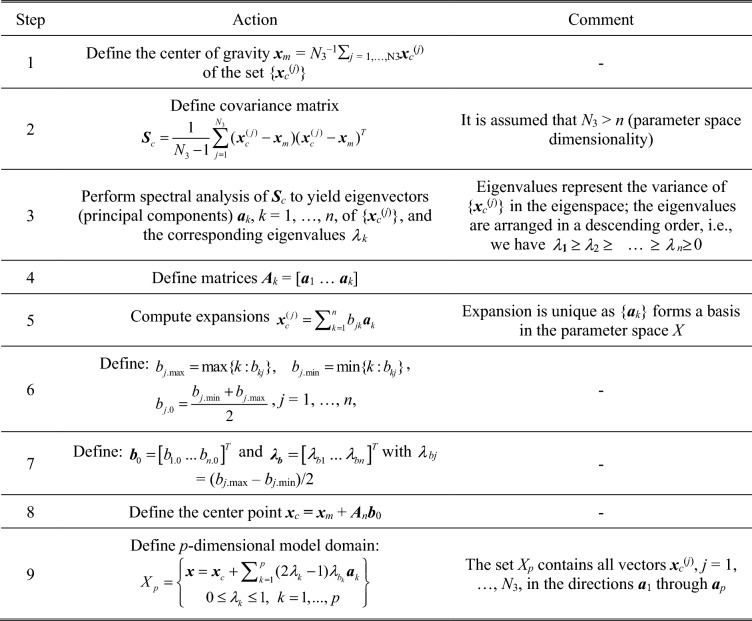
Definition of reduced-dimensionality surrogate model domain.

**Figure 4:**
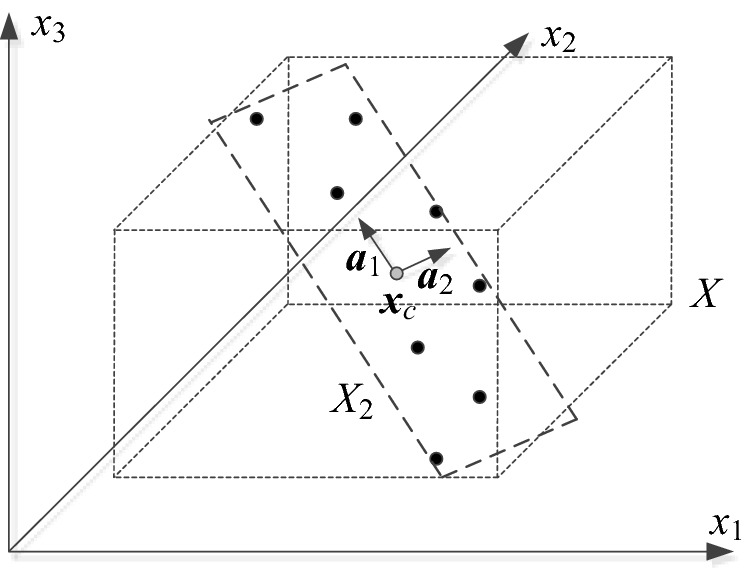
Conceptual illustration of reduced-dimensionality surrogate model domain. Here, a two-dimensional domain *X*_2_ spanned by the two most dominant eigenvectors ***a***_1_ and ***a***_2_; the gray circle represents the center point ***x***_*c*_ (cf. Figure 3).

Figure 3 summarizes the process of defining the surrogate model domain. It follows the procedure proposed in^96^ for domain-confined modelling of high-frequency devices. The main idea is to define the domain of the surrogate model as the smallest set spanned by the most dominant eigenvectors that contains all vectors in {***x***_*c*_^(*j*)^}. In practice, the designs ***x***_*c*_^(*j*)^ are strongly correlated (in the spatial sense), therefore, the dimensionality *p* of the domain can be kept small without losing too much of information. In this work, we keep *p* = 3 for the verification circuits considered in  "Demonstration examples" section. Dimensionality reduction is essential for reducing the number of training data samples (here, denoted as *N*_4_) necessary to build the surrogate model. In this work, we set *N*_4_ = 200, which results in good predictive power of the model (at the level of a few percent of relative RMS error). The training data is obtained from the high-fidelity EM model ***R***_*f*_. Figure 4 provides a graphical illustration of the surrogate model domain definition.

**Figure 5:**
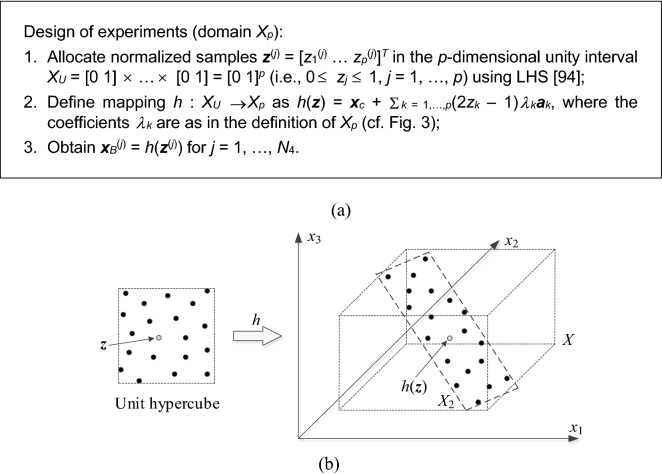
Design of experiments (data sampling) in reduced-dimensionality domain (here, two dimensional): (**a**) sampling procedure, (**b**) graphical illustration: normalized samples are uniformly distributed in the unity interval using LHS, and mapped into *X*_2_ using the transformation *h*.

The surrogate model is constructed using kriging interpolation^54^, although a particular selection of the modeling method is not critical. The training samples, denoted as ***x***_*B*_^(*j*)^ ∈ *X*_*p*_, *j* = 1, …, *N*_4_, are distributed using Latin Hypercube Sampling (LHS)^97^. The design of experiments procedure (cf. Fig. 5) has to account for the fact that the domain is not aligned with the coordinate system axes. The surrogate model will be used to perform global optimization of the circuit within its domain *X*_*p*_.

### Surrogate model optimization for size reduction

The domain of the surrogate model covers the vicinity of the feasible region boundary along the most important directions, as determined using the spectral analysis described in "Surrogate model construction" section (cf. Fig. 3). Having the surrogate, the next stage is to optimize it in a global sense within *X*_*p*_. Due to low dimensionality of the domain, the search process is conducted in two phases:

Exhaustive search on the grid *M*_*p*_ given in the form of a complete set of vectors

7 $$ M_{p}  = \left\{ {\begin{array}{*{20}l}    {{\mathbf{x}} = {\mathbf{x}}_{c}  + \sum\nolimits_{{k = 1}}^{p} {(2\lambda _{k}  - 1)\lambda _{{b_{k} }} {\mathbf{a}}_{k} } } \hfill  \\    {\lambda _{k}  \in \left\{ {0,1/K,2/K, \ldots ,1} \right\},\;\;k = 1, \ldots ,p} \hfill  \\   \end{array} } \right\} $$

where *K* is the grid resolution (we use *K* = 20). The initial design ***x***_*g*_^(0)^ is found by solving

8 $$ {\mathbf{x}}_{g}^{(0)} = \arg \mathop {\min }\limits_{{}} \left\{ {{\mathbf{x}} \in M_{p} \cap X:U({\mathbf{x}})} \right\} $$

Note that ***x***_*g*_^(0)^ is the design that minimizes (surrogate-evaluated) *U* over the intersection of the search grid and parameter space *X* (in general, *X*_*p*_ may extend beyond the original domain *X*);

Local size-reduction-oriented optimization of the surrogate within *X*_*p*_ ∩ *X*, according to (2). The optimization algorithm is a trust-region gradient search described in "Final parameter adjustment" section. For notational simplicity, the design found at this stage will be also denoted as ***x***_*g*_^(0)^.

### Final parameter adjustment

**Figure 6:**
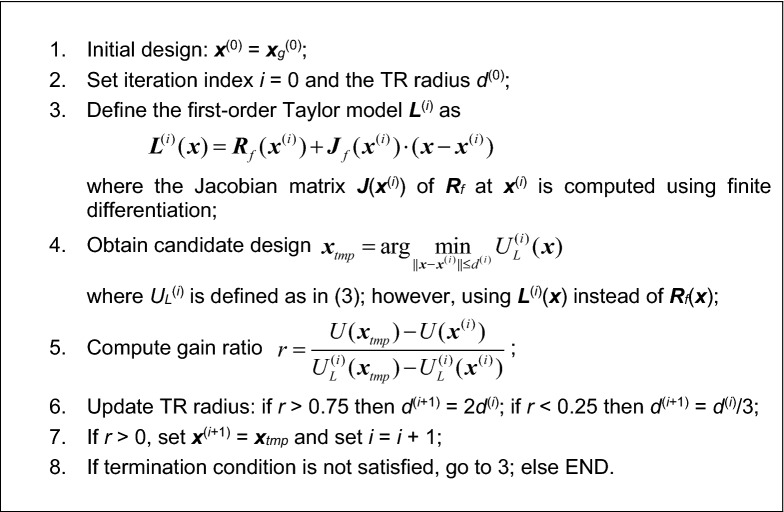
Formulation of the trust-region gradient-based algorithm. The termination condition is based on convergence in argument, ||***x***^(*i*+1)^–***x***^(*i*)^||< *ε*, and reduction of the TR radius, *d*^(*i*)^ < *ε* (whichever occurs first). The termination threshold *ε* is set to 10^−3^ for final tuning of the high-fidelity model, but it is relaxed to 10^−2^ for low-fidelity optimization runs.

The final stage of the global optimization procedure proposed in this paper is a local tuning of the circuit parameter. For accuracy reasons, it is performed at the level of the high-fidelity model ***R***_*f*_. This step is again executed using the trust-region (TR) gradient-based routine^94^, which was also used for initial tuning ("Feasible region boundary approximation" section), and surrogate optimization (" Surrogate model optimization for size reduction" section). The formulation of the TR algorithm has been recalled in Fig. 6.

### Globalized EM-driven size reduction: complete procedure

This section puts together the building blocks of the globalized size reduction algorithm discussed in " Globalized size reduction: explanation of the concept" section through "Final parameter adjustment", and summarizes the operating flow of the entire framework. The algorithm control parameters are gathered in Table 3, their meaning has been already elaborated on earlier. Here we provide general guidelines for their setup. Four parameters of Table 3, i.e., *N*_1_ through *N*_4_, pertain to the computational budget of the entire optimization framework. The number *N*_1_ of samples used for initial approximation of the feasible region boundary is typically set to 500, because, in most practical cases, this value is sufficient and allows for a satisfactory estimation of the said boundary. The next parameter, *N*_2_, i.e., the number of samples for which optimization-based size reduction is carried out, is typically set to 20. This value constitutes a reasonable trade-off between the computational cost of subsequent tuning these deigns and the precision of assessing the surrogate domain. The number *N*_3_ of the refined designs of sufficient quality should somewhat exceed a half *of N*_2_, as this allows for discarding the designs for which the tuning procedure has failed. The fourth parameter controlling the computational budget, i.e., the number *N*_4_ of data samples used for setting up the surrogate model, should be adjusted to ensure the required accuracy of this model (e.g., at the level of a few percent of relative RMS error).

**Table 3:** Control parameters of the proposed globalized size reduction algorithm.

Parameter	Meaning	Recommended value
*N*_1_	The number of random observables generated to obtain initial approximation of the feasible region boundary ("Feasible region boundary approximation" section)	500
*N*_2_	The number of observables selected to conduct size reduction optimization runs at low-fidelity level ("Feasible region boundary approximation" section)	20
*N*_3_	The number of designs selected from the outcome of low-fidelity model optimization runs, and used to define the surrogate model domain ("Feasible region boundary approximation" section)	> $\left\lceil {N_{{2}} /{2}} \right\rceil$
*N*_4_	The number of training data samples for surrogate model construction ("Surrogate model construction" section)	200
*p*	Dimensionality of the surrogate model domain ("Surrogate Model Optimization for Size Reduction" section)	3

**Figure 7:**
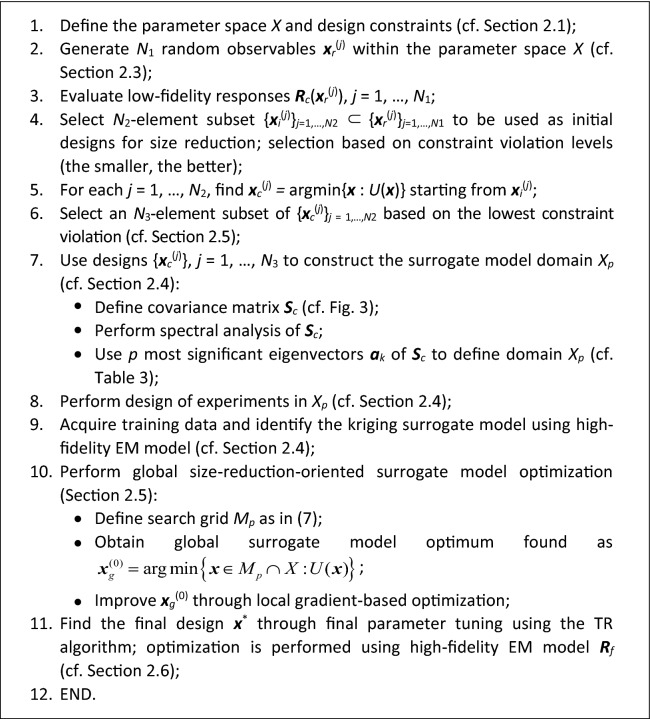
Operating flow of the proposed globalized size reduction algorithm.

**Figure 8:**
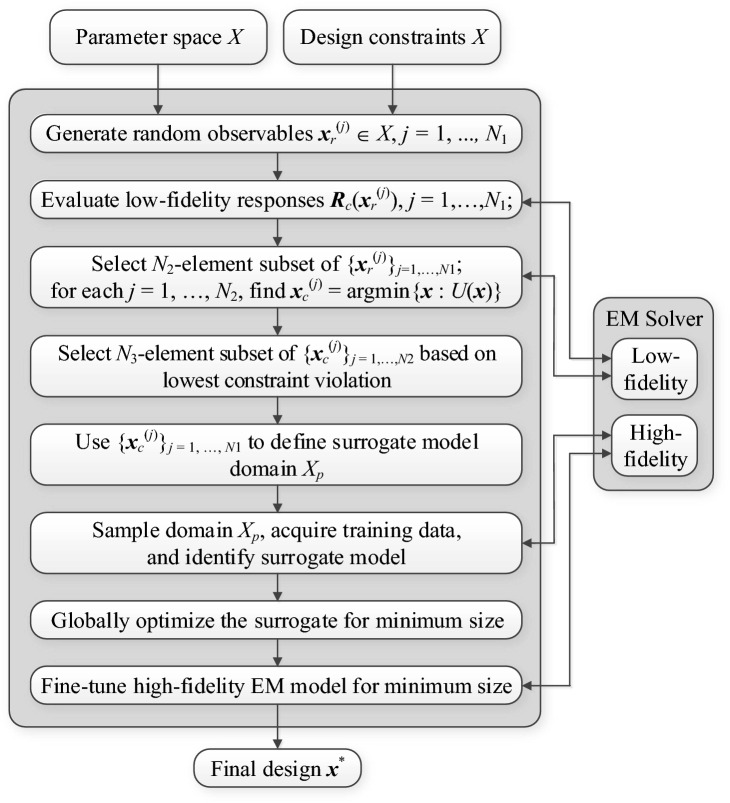
Flow diagram of the proposed globalized size reduction framework.

As for the last parameter *p*, which refers to the surrogate domain dimensionality, it should be kept small (of around one third or half of the number of design variables) to maintain the training data acquisition cost at a reasonable level. The values provided in the Table 3 will be used in the verification experiments of "Demonstration examples" section. The pseudocode of the algorithm can be found in Fig. 7, whereas Fig. 8 shows the flow diagram of the method.

It should also be emphasized that while utilization of the low-fidelity EM model at the early stages of the search process leads to certain inaccuracies (including identification of the feasible region boundary, where the constrained optimum is normally allocated), these are corrected at the final stages, where the high-fidelity EM model is employed to fine-tune the geometry parameters of the circuit.

## Demonstration examples

The proposed globalized size reduction framework is validated with the use of two examples of microstrip circuits, a rat-race coupler (RRC) and a branch-line coupler (BLC). The structures are designed for minimum size, under the constraints imposed on their operating frequency, operating bandwidth, and power split ratio. The performance of the algorithm is compared to nature-inspired optimization using particle swarm optimizer (PSO), as a representative technique of this category, as well as multiple-start gradient search. This remainder of this Section is arranged in a following manner. "Test cases and experimental setup" Section delineates the test cases and the most important experimental settings. "Numerical results" section gathers the numerical results. "Discussion" section contains a discussion that includes qualitative comparisons between the introduced and the benchmark techniques concerning reliability and computational efficiency.

### Test cases and experimental setup

**Figure 9:**
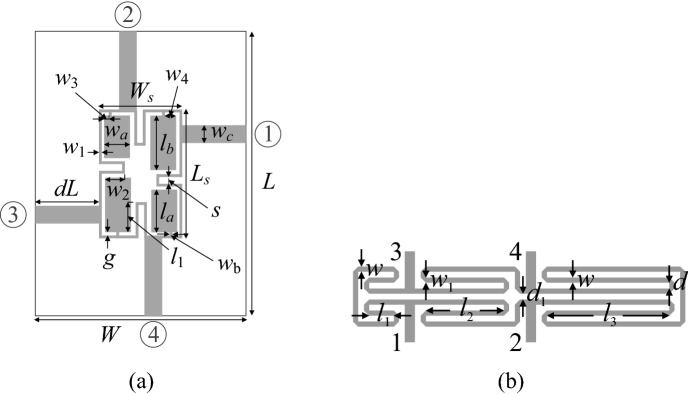
Microstrip structures employed as test cases for verification of the proposed size reduction framework: (**a**) compact branch-line coupler (Circuit I)^98^, (**b**) rat-race coupler with folded transmission lines (Circuit II)^99^.

Verification of the proposed algorithm involves two microstrip circuits, both shown in Fig. 9, and referred to as Circuit I and II, respectively. The evaluation models are rendered in CST Microwave Studio, and simulated with the use of its time-domain solver. The design task is posed as follows:

- Minimize the footprint area *A*(***x***) of the circuit under design;
- Satisfy inequality constraint for matching and port isolation, *g*_1_(***x***) = max{*f* ∈ *F* : max{|*S*_11_(***x***,*f*)|, |*S*_41_(***x***,*f*)|}} + 20 dB;
- Satisfy equality constraint for the power split ratio: *h*_1_(***x***) =| |*S*_31_(***x***,*f*_0_)|–|*S*_21_(***x***,*f*_0_)| |= 0 (both transmission responses are in dB);

The first constraint corresponds to a condition that both |*S*_11_(***x***,*f*)| and |*S*_41_(***x***,*f*)| should not be greater than − 20 dB over the operating band *F*. The second constraint requires the circuit to maintain an even power split ratio at its operating frequency *f*_0_. The objective function is formulated as in (3) with the penalty functions defined as in Tables 1 and 2. Table 4 provides essential parameters for both circuits, including design variables, parameter spaces, operating frequencies, etc.

**Table 4:** Essential parameters of Circuits I and II of Fig. 9.

Circuit	I^98^	II^99^
Substrate	AD300 (*ε*_*r*_ = 2.97, *h* = 0.76 mm)	RO4003 (*ε*_*r*_ = 3.38, *h* = 0.762 mm)
Designable Parameters [mm]	***x*** = [*g l*_1*r*_* l*_*a*_* l*_*b*_* w*_1_ *w*_2*r*_* w*_3*r*_* w*_4*r*_* w*_*a*_* w*_*b*_]^*T*^	***x*** = [*l*_1_ *l*_2_ *l*_3_ *d w w*_1_]^*T*^
Other Parameters [mm]	*L* = 2*dL* + *L*_*s*_, *L*_*s*_ = 4*w*_1_ + 4* g* + s + *l*_*a*_ + *l*_*b*_, *W* = 2*dL* + *W*_*s*_, *W*_*s*_ = 4*w*_1_ + 4* g* + *s* + 2*w*_*a*_, *l*_1_ = *l*_*b*_*l*_1*r*_, w_2_ = *w*_*a*_*w*_2*r*_, *w*_3_ = *w*_3*r*_*w*_*a*_, and *w*_4_ = *w*_4*r*_*w*_*a*_, *w*_*c*_ = 1.9 mm	*d*_1_ = *d* +|*w*–*w*_1_|, *d* = 1.0, *w*_0_ = 1.7, and *l*_0_ = 15 mm
Parameter space *X*	***l*** = [0.4 0.1 3.0 3.0 0.4 0.1 0.1 0.1 2.0 0.2]^*T*^ ***u*** = [1.0 0.99 15.0 25.0 1.5 0.99 0.9 0.9 12.0 1.0]^*T*^	***l*** = [0.1 5.0 5.0 0.2 0.2 0.5]^*T*^ ***u*** = [15.0 30.0 50.0 2.0 2.0 2.0]^*T*^
Operating parameters	*f*_0_ = 1.5 GHz *F* = [1.45 1.55] GHz	*f*_0_ = 1.0 GHz *F* = [0.95 1.05] GHz
Low-fidelity EM model	~ 24,000 mesh cells Simulation time 110 s	~ 50,000 mesh cells Simulation time 55 s
High-fidelity EM model	~ 160,000 mesh cells Simulation time 240 s	~ 200,000 mesh cells Simulation time 160 s

The low-fidelity models of both verification circuits are obtained by reducing discretization density of the structure. The proportion of simulation times between the high- and low-fidelity model is 2.2 and 2.9 for Circuit I and II, respectively, which will carry over to computational savings of the entire optimization procedure.

It should be emphasized that the search spaces are large in terms of the ranges of geometry parameters (average upper-to-lower bound ratio is almost seven in the case of Circuit I and over thirty for Circuit II). Furthermore, both circuits feature parameter redundancy, i.e., additional variables related to the specific circuit geometries (utilization of CMRCs for Circuit I, and transmission line meandering for Circuit II). Both factors make the design tasks multimodal, in particular, size reduction outcome will very much depend on the initial design. At the same time, global search methods are likely to exhibit limited repeatability of solutions due to the parameter space dimensionality and overall size. In order to take this into account, verification experiments are carried out in a statistical sense, by running multiple instances of the proposed and benchmark algorithms, and comparing statistical moments of the outcomes. More specifically, each algorithm is run ten times. The figures of interest to be compared are average circuit size along with the standard deviation of the size, as well as average violation of design constraints (and the corresponding standard deviations). Another factor to be compared is the computational cost of the optimization process. Table 5 briefly outlines the two benchmark methods utilized in this work, multiple-start gradient search, and the particle swarm optimizer (PSO).

**Table 5:** Benchmark algorithms.

Algorithm	Description
I	Local gradient-based size reduction using the trust region algorithm (cf. "Final parameter adjustment" section). The optimization problem is formulated as in (2), (3)
II	Particle swarm optimizer (PSO)^100^, employed as a representative nature-inspired technique. The algorithm setup is as follows: swarm size of 10, maximum number of iterations 100, standard setup of control parameters (*χ* = 0.73, *c*_1_ = *c*_2_ = 2.05), cf.^100^. The problem formulated as in (2), (3)

The reason for incorporating gradient search is to demonstrate multi-modality of the considered design tasks. On the other hand, PSO is employed to verify whether the proposed algorithm is capable to bring any advantages over nature-inspired procedures, both in terms of computational efficiency and design quality. Note that the computational budget of PSO has been limited to 1000 EM simulations, which is clearly insufficient from numerical perspective, yet this number can be considered borderline from the perspective of practicality: even for relatively low-cost computational models of Circuit I and II, the PSO runs take a few days each.

### Numerical results

**Figure 10:**
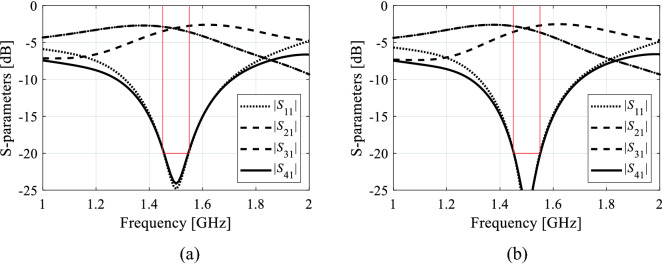
Circuit I: EM-simulated scattering parameters for two selected designs obtained using the proposed size reduction algorithm: (**a**) design 1 (footprint area 305.1 mm^2^), (**b**) design 2 (footprint area 302.4 mm^2^). Target operating frequency and bandwidth indicated using the vertical and horizontal lines, respectively.

**Figure 11:**
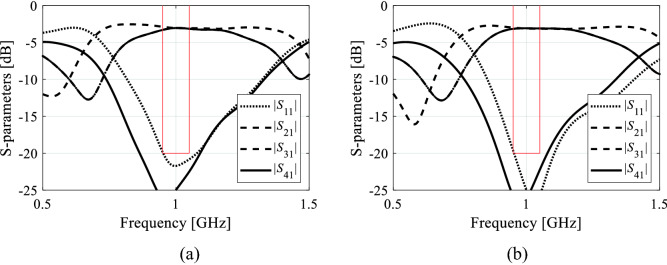
Circuit II: EM-simulated scattering parameters for two selected designs obtained using the proposed size reduction algorithm: (**a**) design 1 (footprint area 370 mm^2^), (**b**) design 2 (footprint area 364 mm^2^). Target operating frequency and bandwidth indicated using the vertical and horizontal lines, respectively.

The results obtained for the proposed framework and the benchmark algorithms have been gathered in Tables 6 and 7 for Circuit I and II, respectively. Figures 10 and 11 show the circuit *S*-parameters at the final designs found during the selected runs of the proposed procedure. As mentioned earlier, the data contains the mean values of the circuit size, violations of the inequality and equality constraints, as well as standard deviations thereof, all computed over the ten runs of each algorithm. The mean figures can be viewed as performance metrics, whereas standard deviations quantify the repeatability of solutions.

**Table 6:** Optimization results for Circuit I.

Optimization algorithm	Performance figure						
	Circuit size *A* [mm^2^] ^1^	Std(*A*) ^2^	Inequality constraint		Equality constraint		CPU cost^7^
			Violation *D*_1_ [dB] ^3^	Std(*D*_1_) [dB] ^4^	Violation *D*_2_ [dB] ^5^	Std(*D*_2_) [dB] ^6^	
Algorithm I	295.1	24.7	3.6	1.9	0.2	0.1	77 × ***R***_*f*_ [5.2 h]
Algorithm II	541.5	240.4	5.5	6.8	0.7	0.1	1,000 × ***R***_*f*_ [66.7 h]
Globalized search with dimensionality reduction (this work)	301.8	3.9	0.4	0.2	0.1	0.03	852 × ***R***_*f*_ [56.8 h]

^1^ Optimized footprint area of the circuit averaged over ten algorithm runs.

^2^ Standard deviation of the optimized footprint area averaged over ten algorithm runs.

^3^ Violation of inequality constraint, defined as *D*_1_ = max{*f* ∈ *F* : max{|*S*_11_(***x***,*f*)|, |*S*_41_(***x***,*f*)|}} + 20 dB, averaged over ten algorithm runs.

^4^ Standard deviation of the constraint violation *D*_1_, averaged over ten algorithm runs.

^5^ Violation of equality constraint, defined as *D*_2_ =| |*S*_31_(***x***,*f*_0_)|–|*S*_21_(***x***,*f*_0_)| | dB, averaged over ten algorithm runs.

^6^ Standard deviation of the constraint violation *D*_2_, averaged over ten algorithm runs.

^7^ Cost expressed in terms of equivalent number of high-fidelity EM analyzes. Numbers in brackets correspond to the running time in hours.

**Table 7:** Optimization results for Circuit II.

Optimization algorithm	Performance figure						
	Circuit size *A* [mm^2^]^1^	Std(*A*) ^2^	Inequality constraint		Equality constraint		CPU cost^7^
			Violation *D*_1_ [dB] ^3^	Std(*D*_1_) [dB] ^4^	Violation *D*_2_ [dB] ^5^	Std(*D*_2_) [dB] ^6^	
Algorithm I	378.0	59.3	4.5	4.3	0.2	0.2	63 × ***R***_*f*_ [2.8 h]
Algorithm II	543.1	86.8	− 1.0	1.6	0.1	0.1	1000 × ***R***_*f*_ [44.4 h]
Globalized search with dimensionality reduction (this work)	370.7	20.8	0.0	0.8	0.1	0.05	584 × ***R***_*f*_ [25.9 h]

^1^ Optimized footprint area of the circuit averaged over ten algorithm runs.

^2^ Standard deviation of the optimized footprint area averaged over ten algorithm runs.

^3^ Violation of inequality constraint, defined as *D*_1_ = max{*f* ∈ *F* : max{|*S*_11_(***x***,*f*)|, |*S*_41_(***x***,*f*)|}} + 20 dB, averaged over ten algorithm runs.

^4^ Standard deviation of the constraint violation *D*_1_, averaged over ten algorithm runs.

^5^ Violation of equality constraint, defined as *D*_2_ =| |*S*_31_(***x***,*f*_0_)|–|*S*_21_(***x***,*f*_0_)| | dB, averaged over ten algorithm runs.

^6^ Standard deviation of the constraint violation *D*_2_, averaged over ten algorithm runs.

^7^ Cost expressed in terms of equivalent number of high-fidelity EM analyzes. Numbers in brackets correspond to the running time in hours.

### Discussion

The performance analysis of the proposed algorithm, and the comparison with the benchmark methods will be carried out using the results contained in Tables 6 and 7. One can formulate the following observations:

- The results obtained using Algorithm I (multiple-start gradient-based optimizer) demonstrate that the considered design problems are indeed multimodal. The standard deviation of the footprint area is close to ten percent of the average area (Circuit I), and it exceeds fifteen percent (Circuit II). This means that the optimization results are highly dependent on the initial design, which—in turn—indicates the need for global search. It should also be noted that although Algorithm I produces designs that exhibit small size on the average, the constraint control is poor. In particular, a typical violation of the first constraint is around four decibels.
- The performance of nature-inspired optimization (here, using PSO) is poor. The circuit sizes achieved with Algorithm II are significantly larger than for the remaining methods with high standard deviation. Also, constraint control is inferior and inconsistent between the algorithm runs. These results are partially associated with a limited computational budged assigned for Algorithm II (1000 objective function evaluations). It appears that achieving usable results would require significantly larger budgets, probably at the level of 5000 to 10,000 EM simulations, which is not practical.
- The proposed algorithm exhibits the best consistency out of the entire benchmark set. The average circuit size is small (and comparable with Algorithm I); however, the average constraint violations are much smaller (only 0.4 dB and 0.0 dB for the first constraint, and 0.1 dB for the second constraint, on the average). At the same time, the standard deviation of the circuit area is considerable lower than for the benchmark methods: it is only about 1.3 percent (in relation to the average size) in the case of Circuit I, and only about five percent in the case of Circuit II. This corroborates truly global search capabilities of the presented method.
- Computational overhead of the presented algorithm is clearly much higher than that of local optimization, yet it is lower than for Algorithm II. As mentioned earlier, achieving reasonable results with the PSO algorithm would require increasing its computational budget by a factor five to ten, which means that the cost of the proposed algorithm can be estimated as one order of magnitude lower than for the nature-inspired methods.

The overall efficacy of the proposed size reduction procedure is superior over the benchmark. Within reasonable computational budget, the algorithm produces consistent results in terms of the circuit footprint areas with remarkably low standard deviation over the set of repetitive runs. At the same time, it exhibits excellent control of the design constraints: the average violations are around a small fraction of a decibel. Competitive computational cost is a result of employing variable-resolution EM models but also due to dimensionality reduction at the stage of constructing the surrogate model for globalized search stage of the optimization process.

## Conclusion

In this work, we introduced a technique for EM-driven miniaturization of passive microwave components. The foundation of the presented methodology is parameter pre-screening and initial optimization runs (both carried out using low-fidelity simulation model), oriented towards identification of the special location of the feasible region boundary. The reduced-dimensionality surrogate model established in this region is employed to perform global size reduction, followed by gradient-based parameter tuning. The last two stages are executed using high-fidelity EM model for reliability reasons. The combination of the developed algorithmic approaches results in an optimization framework that enables globalized size reduction at low computational expenses. Comprehensive validation involving two microstrip couplers corroborates the efficacy of the proposed technique, and its superiority over local (gradient-based) parameter tuning as well as nature-inspired optimization, here, represented by the particle swarm optimization algorithm. The numerical results demonstrate global search capability, as well as consistent results, both in terms of the achieved circuit footprint, constraint control, and the computational cost. The latter is a consequence of the implemented mechanisms, i.e., dimensionality reduction and variable-fidelity EM simulations. One of the objectives of the future work will be to improve the feasible region boundary identification stage of the algorithm, as well as extending the range of applicability to include a larger variety of microwave components and antenna structures.

## Data Availability

The datasets generated during and/or analysed during the current study are available from the corresponding author on reasonable request. Contact person: anna.dabrowska@pg.edu.pl.
